# Efficacy of sulphadoxine-pyrimethamine with or without artesunate for the treatment of uncomplicated *Plasmodium falciparum *malaria in southern Mozambique: a randomized controlled trial

**DOI:** 10.1186/1475-2875-8-141

**Published:** 2009-06-26

**Authors:** Elizabeth N Allen, Francesca Little, Tunisio Camba, Yasmin Cassam, Jaishree Raman, Andrew Boulle, Karen I Barnes

**Affiliations:** 1 Division of Clinical Pharmacology, Department of Medicine, University of Cape Town, South Africa; 2 Department of Statistical Sciences, University of Cape Town, South Africa; 3 Ministry of Health, Mozambique; 4 Malaria Research Lead Programme, Medical Research Council, Durban, South Africa; 5 School of Public Health and Family Medicine, University of Cape Town, South Africa

## Abstract

**Background:**

An artemisinin-based combination therapy, artesunate (AS) plus sulphadoxine-pyrimethamine (SP), was compared to SP monotherapy to provide evidence of further treatment options in southern Mozambique.

**Methods:**

Between 2003 and 2005, 411 patients over one year and 10 kg with uncomplicated *Plasmodium falciparum *malaria were randomly allocated SP (25/1.25 mg per kg day 0) or AS/SP (as above plus 4 mg/kg artesunate days 0, 1 and 2). Allocation was concealed, but treatment was open-label except to microscopists. The primary objective was the relative risk of treatment failure, which was assessed using World Health Organization response definitions modified to a 42-day follow-up.

**Results:**

Of the 411 subjects enrolled, 359 (87.3%) completed the follow up period (SP n = 175, AS/SP n = 184). A survival analysis including 408 subjects showed that the polymerase chain reaction-adjusted cure rates were 90.4% (95% confidence interval [CI] 84.9%–93.9%) and 98.0% (95% CI 94.8%–99.3%) for SP and AS/SP respectively. Multivariable analysis showed that treatment with AS/SP decreased the relative hazard of treatment failure by 80% compared to SP (hazard ratio [HR] 0.2; 95% CI 0.1–0.6) and age over seven years decreased the relative hazard of failure by 70% (HR 0.3; 95% CI 0.1–0.9), when compared to younger age. However, having a quintuple *dhfr*/*dhps *mutation increased the relative hazard of failure compared to fewer mutations (HR 3.2; 95% CI 1.3–7.5) and baseline axillary temperature increased the relative hazard of failure by 50% for each °C increase (HR 1.5; 95% CI 1.1–2.2).

**Conclusion:**

While both treatments were efficacious, AS plus SP significantly decreased the relative hazard of treatment failure compared to SP monotherapy Artesunate plus sulphadoxine-pyrimethamine, but not sulphadoxine-pyrimethamine monotherapy, met the current WHO criteria of >95% efficacy for policy implementation.

**Trial registration:**

NCT00203736 and NCT00203814

## Background

Mozambique (population *circa *20,000,000) is a poor country with a life expectancy of 45 years. Malaria is responsible for a large health burden, including an estimated 19% of deaths among children under five years of age [1]. Transmission intensity of the predominantly *Plasmodium falciparum *malaria varies throughout the country according to the season, other environmental factors, and the use of vector control measures. A treatment policy with effective anti-malarials is a key component of malaria control programmes and data regarding resistance of parasites to anti-malarial drugs are now a critical factor in drug policy decision-making. While chloroquine was the national policy in Mozambique for the treatment of uncomplicated malaria as recently as 2003, evidence of its poor efficacy throughout Africa since the 1980s signalled a need for alternative anti-malarials [2]. Artemisinin-based combination therapy (ACT) is now generally considered the best treatment for uncomplicated *falciparum *malaria provided the partner drug is efficacious. ACT deliverers a more rapid cure compared to non-ACT, reducing gametocyte carriage (the parasites' sexual stage, thereby reducing infectivity), and delaying the development of anti-malarial resistance [3-5]. The artemisinin derivative, artesunate (AS), is particularly effective in the rapid treatment of uncomplicated malaria, and its use in combination with the longer-acting sulphadoxine-pyrimethamine (SP) is currently one of four forms of ACT recommended by the World Health Organization (WHO) [6,7]. From a programmatic perspective, AS plus SP has the unique advantage of the full dose of the partner drug being administered under supervision when malaria is diagnosed.

There was clear evidence that ACTs substantially reduced treatment failure, recrudescence and gametocyte carriage but this had not been reported for Mozambique [8]. However, amodiaquine plus SP was selected as the national treatment policy for uncomplicated *falciparum *malaria due to its lower cost. At this time the Ministry of Health recommended that a phased implementation of an ACT be studied in parallel in Maputo Province. Efficacy of SP monotherapy had been assessed by the South East African Combination Anti-malarial Treatment (SEACAT) evaluation in Namaacha and Matatuine Districts, Maputo Province, during 2002 and data showed that a combined polymerase chain reaction (PCR)-adjusted cure rate exceeded the 80% level considered for it to be combined with other anti-malarials to sustain its useful therapeutic life [7,9]. The prevalence of the quintuple dihydrofolate reductase (*dhfr*) and dihydropteroate synthetase (*dhps*), mutation across all study sites was 5–6% throughout the study period [[10], Raman J personal communication]. This randomised controlled trial (RCT) was conducted during the malaria seasons between 2003 and 2005 to compare SP with AS plus SP, in order to provide evidence to health policy makers of further treatment options.

## Methods

### Study location and design

**Figure 1:**
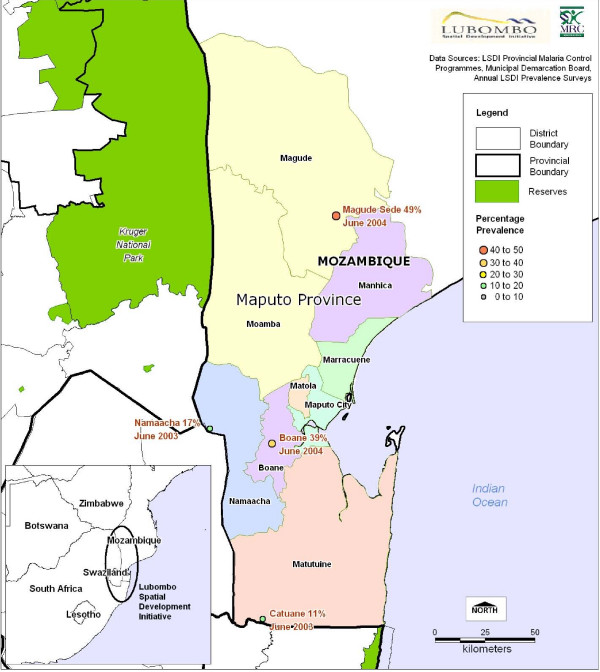
**Geographical location of the 4 study sites in Maputo Province showing prevalence of malaria at the study start**.

This multi-centre, open-label, parallel-group RCT was conducted in Maputo Province, Southern Mozambique in four public-sector health facilities in two phases: initially Catuane and Namaacha (2003), thereafter Boane and Magude (2004–2005) (Figure 1). There had been a marked decline in malaria prevalence throughout Maputo Province from 1999 until the time of the study start (Figure 1). This is attributed to an intensive intervention of indoor residual spraying (IRS) [11].

### Study subjects

Male and non-pregnant, non-breastfeeding female patients with malaria symptoms, that were older than one year of age and weighed more than 10 kg, were invited to give informed consent prior to screening for *P. falciparum *infection by rapid diagnostic test (Immunochromatographic Test, P.f^®^., SA Scientific). Patients diagnosed with *P. falciparum *parasitaemia up to 500,000 asexual parasites/μml blood (on thick Giemsa-stained smear) and with axillary temperature greater than or equal to 37.5°C (or history of fever within the previous 24 hours) were suitable for inclusion. Patients receiving folate or anti-malarials (including antibiotics with anti-malarial properties not used for malaria) within seven days were excluded. Also excluded were patients with danger signs (prostration, repeated vomiting and/or dehydration) or severely ill, those with a history of G6PD deficiency or allergy to the study (or related) drugs, or those with serious underlying disease [12,13].

### Study treatments, enrolment and follow up

Eligible subjects were randomized to treatment with SP monotherapy (Fansidar^®^. Roche, Gauteng, South Africa) or AS/SP (co-packaged as Arsudar^®^. Sanofi, Gauteng, South Africa) (Table 1). Subjects were observed until an hour post-dose in case a repeat dose was required for vomiting. A computerized random allocation schedule was generated for each site divided sequentially into the three weight-based dose levels, within which there was an equal chance of a subject receiving SP or AS/SP. Treatment allocation was concealed from site staff using opaque padded, sealed envelopes. Subsequently treatments were open-label, except to microscopists who remained blinded to treatment allocation.

**Table 1:** Numbers of artesunate 50 mg tablets (4 mg/kg) and sulphadoxine-pyrimethamine 500 mg/25 mg tablets (25/1.25 mg/kg) for each weight group

Mass (kg)	*Approximate age (years)*	*No. of SP tablets (all subjects)*	*No. of AS tablets (AS/SP subjects)*		
		
		*Day 0*	*Day 0*	*Day 1*	*Day 2*
10–20	1 – 6	1	1	1	1
	
21–35	7 – 13	2	2	2	2
	
**> 35**	14 +	3	4	4	4

SP: sulphadoxine-pyrimethamine

AS/SP: artesunate/sulphadoxine-pyrimethamine

The visit schedule was based on the WHO protocol for assessment of *in vivo *therapeutic efficacy of anti-malarials, with extension to a 42 day follow-up due to the long elimination half-life of SP [14,15]. After enrolment on day 0, subjects were asked to return on days 1, 2, 3, 7, 14, 21, 28 and 42 for assessment of axillary temperature by electronic thermometer, asexual parasite density (by thick Giemsa-stained smear from finger-prick sample), haemoglobin concentration (by finger prick sample, HemoCue^®^), clinical signs and symptoms to capture adverse events, and concomitant medications [16]. A finger prick sample of blood blotted on filter paper (Whatman No. 1) was also taken at each visit for molecular analyses. Subjects were considered lost to follow up after three attempts to contact them by the study team.

### Laboratory investigations

Asexual parasite density was calculated using the number of asexual parasites and assuming 8,000 leukocytes/μl blood. Genotyping of *P. falciparum *DNA extracted from these dried samples based on variations in three highly variable proteins (merozoite surface proteins 1 and 2, and glutamine-rich protein) was used to determine if treatment failure was due to a re-infection or recrudescence of the original infection [17,18]. Infections were classified as recrudescent if PCR products for all three markers from day 0 and day of failure parasites were identical. If the banding patterns for any markers differed between day 0 and day of failure parasites, the infection was classified as a re-infection. Polymorphisms in the dihydrofolate reductase, *dhfr *(codons 51, 59, 108, 164) and dihydropteroate synthetase, *dhps *(codons 436, 437, 540 and 581) genes were detected using nested PCR and restriction endonuclease cleavage [19]. Digestion products separated on a 2% agarose gel using electrophoresis were visualized and photographed using a MiniBIS documentation system. Genotype of each codon was classified as pure wild, pure mutant or mixed (both mutant and wild alleles in one sample).

### Data management and analytical methods

The primary study objective was to compare the risk of treatment failure between the treatment groups, adjusted for baseline characteristics. A secondary outcome compared the rate of parasite clearance between groups.

Response to treatment was classified according to WHO definitions for low to moderate malaria transmission intensity areas, modified for a 42-day follow up [14,15]. Subjects with clinical or parasitological failure, clinical deterioration other than treatment failure, an adverse event (AE) requiring treatment withdrawal, allergic reaction during the treatment course, or concomitant prescribing of contra-indicated drugs, were withdrawn and given rescue treatment with quinine as necessary.

Key fields were verified by a study monitor and double-entered into an MS Access 2000 (Microsoft Corporation, Seattle, USA) database. Laboratory data were recorded electronically. Data were imported into Stata/IC 10.0 (StataCorp LP, College Station, Texas, USA) when outcomes, time-to-event indicators and explanatory variables were programmed.

Major protocol violations were defined as subjects: i) missing days 1, 2, 3 of the ACT arm or days 2,3 of the monotherapy arm; ii) taking folic acid or a concomitant medication with anti-malarial activity (cotrimoxazole, trimethoprim, chloramphenicol and tetracyclines, with erythromycin added retrospectively to this list); iii), whose day 42 visit was more than three days late; and iv) taking an incorrect dose or repeat dose for vomiting. Data were not censored for missed visits as it was assumed intensive AE monitoring would detect malaria symptoms or treatment. Missing data fields were dropped where appropriate and subjects lost to follow-up after day 0 were automatically dropped from the time-to-event analyses. An attempt was made to follow all subjects to day 42 despite protocol violations.

Sample sizes were calculated assuming an adequate clinical and parasitological response (ACPR) of 75% for SP and 90% for AS/SP (confidence interval [CI] 95% and 80% power) indicating 100 per treatment group in each phase of the study (400 subjects in total).

Time-to-event outcomes, which were considered the most appropriate for the data, were analysed using Kaplan Meier (KM) survival methods and Cox's Proportional Hazards Regression. This allowed data up to the point of loss to follow-up or withdrawal to be included and for the relative hazard to approximate the relative risk.

KM distributions and survival curves for the two treatment groups were compared using a log rank test. Should model diagnostics suggest hazards were not proportional, parametric models were applied [20]. Time to treatment failure was defined as time from day 0 until day of failure due to recrudescence (early treatment failure [ETF], late clinical failure [LCF] or late parasitological failure [LPF]). Time to parasite clearance was time from day 0 to the first of two consecutive zero parasite density readings. The analyses were repeated after excluding subjects who had dose-related protocol violations and censoring the follow-up for all other protocol violations at the time of their occurrence.

### Ethical issues

The study was approved by the Ethics Committees of the Mozambican Ministry of Health and the University of Cape Town prior to commencement, and conducted in accordance with the South African Clinical Trials Guidelines 2000 [21]. Staff members were trained in Good Clinical Practice including how to request informed consent in the local language. Illiterate patients marked 'X' in the presence of an independent literate witness who signed the consent form. Any serious adverse events were reported to both ethics committees.

## Results

**Figure 2:**
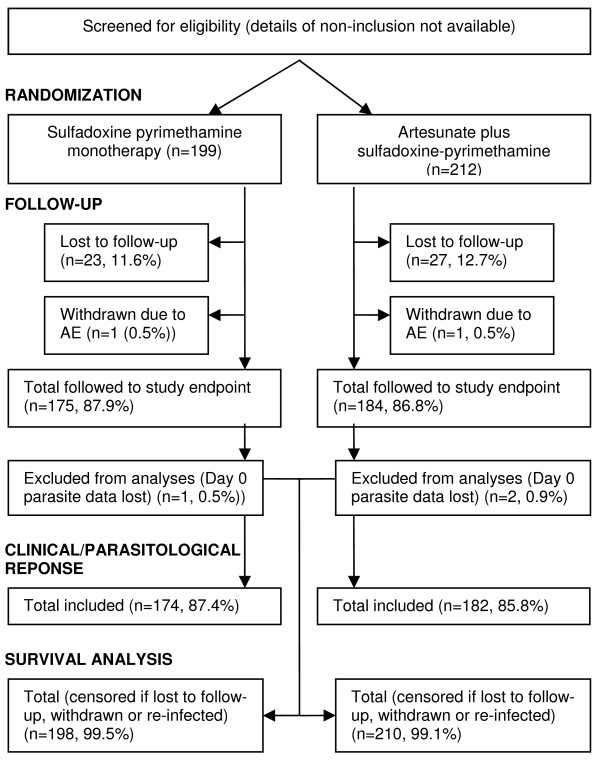
**Subject disposition and the analysis dataset**.

Subject disposition is shown in Figure 2. Of 411 subjects randomized to SP or AS/SP, 359 (87.3%) were followed to assessment of the study outcome (SP: 175 [87.9%], AS/SP 184 [86.8%]). The main reason for loss to follow-up was reportedly movement from the study area, while one subject in each treatment group was withdrawn for an AE. There were no differences in baseline and clinical characteristics between treatment groups (Table 2) and protocol violations were also similar. Three subjects positive according to rapid diagnostic test and who completed the study were removed from all analyses as their day 0 slides were lost prior to parasite density confirmation. Of the remaining subjects, 27 (SP: 18 [10.3%], AS/SP: 9 [4.9%] p = 0.054) were rescued for re-treatment of malaria, but were found by PCR to have been re-infected. Excluding these re-infections, 138 (88.5%) in the SP arm and 169 (97.7%) in the AS plus SP arm achieved an ACPR (P = 0.0008). The proportion of ETFs was similar between the two treatment arms but there were significant differences for all other response categories, including re-infections (Table 3).

**Table 2:** Baseline and clinical characteristics of all subjects by treatment

**Continuous variables**	SP (n = 199)	AS/SP (n = 212)	Total (n = 411)	p-value
Haemoglobin (g/dL) (mean/SD)	11.0 2.1)	11.4 (2.1)	11.2 (2.1)	0.07*
Temperature (°C) (mean/SD)	37.6 (1.2)	37.4 (1.2)	37.5 (1.2)	0.251*
Dose of pyrimethamine (mg/kg) (mean/SD)	1.7(0.7)	1.8 (0.6)	1.7 (0.6)	0.645*
Dose of artesunate (mg/kg) (mean/SD)	N/a	11.4 (3.0)	11.4 (3.0)	N/a
Age (years) (median/IQR)	10.0 (5.0–22.0)	12.0 (5.0–24.0)	11.0 (5.0–23.0)	0.237#
Weight (kg) (median/IQR)	25.0 (15.0–52.0)	28.0 (15.0–55.0)	25.8 (15.0–54.0)	0.508#
Parasite density (per μl) (geometric mean/95% CI)	3630.7 (2464.8; 5348.1)	2885.2 (2028.4; 4104.0)	3225.6 (2486.3; 4184.8)	0.284#
Log parasite density (per μl) (median/IQR)	3.8 (2.5–4.5)	3.6 (2.5–4.5)	3.7 (2.5–4.5)	0.315#
Duration malaria symptoms (days) (median/IQR)	1.0 (1.0–3.0)	2.0 (1.0–3.0)	2.0 (1.0–3.0)	0.347#
**Categorical variables (n/%)**				
Gender male	76 (38)	94 (44)	170 (41)	0.206^
Age 7 years or under	84 (42.2)	79 (37.3)	163 (39.7)	0.306^
Anaemic (haemoglobin < 11 g/dL)^1^	94 (47)	94 (44)	188 (46)	0.556^
Severely anaemia (haemoglobin < 7 g/dL)^1^	6 (3)	2 (1)	8 (2)	0.129^
History of vomiting	20 (10)	17 (8)	37 (9)	0.472^
History of vomiting, with fever on day 0	9 (5)	6 (3)	15 (4)	0.361^
Vomited within 1 hour of any dose	7 (4)	12 (6)	19 (5)	0.301^
Diarrhoea in first 24 hrs post dose	14 (7)	7 (3)	21 (5)	0.086^
Diarrhoea between days 2 and 7	7 (4)	5 (2)	12 (3)	0.485^
Quintuple mutation	45/195 (23.1)	39/210 (18.6)	84(20)	0.289^

SP: sulphadoxine-pyrimethamine

AS/SP: artesunate/sulphadoxine-pyrimethamine

^1 ^WHO 2001

N/a Not applicable

* Student's two sample t-tests

#Wilcoxon rank sum test

^Chi-square or Fisher's exact tests

**Table 3:** Clinical and parasitological response by treatment group for subjects completing follow up

*Response* *n(%)*	*SP* *N = 174*	*AS/SP* *N = 182*	*Total* *N = 356*	P*
Re-infection	18 (10.3)	9 (4.9)	27 (7.6)	0.054

Success (ACPR)	138 (88.5)	169 (97.7)	307 (93.3)	0.0008
Treatment failure	18 (11.5)	4 (2.3)	22 (6.7)	0.0008
ETF	5 (3.2)	3 (1.7)	8 (2.4)	0.39
LCF	4 (2.6)	0	4 (1.2)	0.03
LPF	9 (5.8)	1 (0.6)	10 (3.0)	0.006

As patients with re-infection were withdrawn and rescued prior to study completion they are excluded form denominators for ACPR and treatment failure

SP: sulphadoxine-pyrimethamine

AS/SP: artesunate/sulphadoxine-pyrimethamine

ACPR: adequate clinical and parasitological response

ETF: early treatment failure

LCF: late clinical failure

LPF: late parasitological failure

*2-sample test of proportions

**Figure 3:**
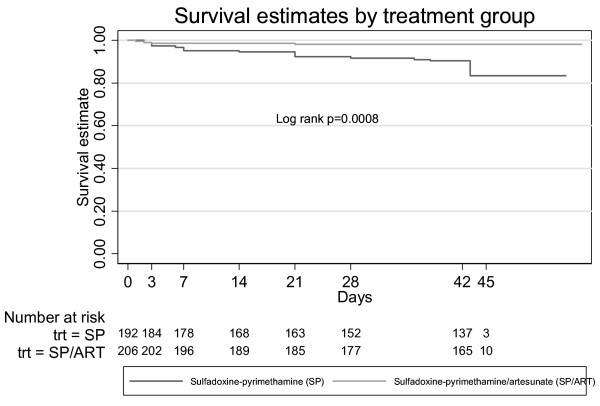
**Kaplan-Meier survival curves for time to treatment failure**.

From the survival analysis, Figure 3 illustrates that ACPR rates were consistently higher in the ACT group compared to the SP monotherapy group (log-rank p-value = 0.0008). Day 42 success rates were 90.4% (95% CI 84.9%–93.9%) for those taking SP monotherapy and 98.0% (95% CI 94.8%–99.3%) for those taking the ACT. The multivariate analysis (Table 4) showed the ACT decreased the relative hazard of failure by 80% compared to monotherapy, while age over seven years decreased the relative hazard of failure by 70% compared to younger age, baseline axillary temperature on Day 0 increased the relative hazard of failure by 50% for each additional 1°C, and a quintuple *dhfr*/*dhps *mutation increased the relative hazard of failure 3.2 fold compared to fewer mutations. The proportional hazard assumption was satisfied overall; however there was an accelerated time to failure (approximately 6-fold) for subjects with a quintuple mutation compared to subjects with fewer mutations suggesting that early treatment failure is particularly associated with presence of a quintuple mutation. When the analysis was repeated after excluding or censoring for major protocol violations (subjects included; SP n = 177 AS/SP n = 177), results confirmed those found above.

**Table 4:** Multivariate model for the relative risk of treatment failure

Variable	*Hazard ratio*	*95% CI*	*P*
Treatment with AS/SP vs SP	0.2	0.1–0.6	0.004
Age > 7 years vs. age ≤ 7 years	0.3	0.1–0.9	0.033
Presence of a quintuple mutation vs. fewer mutations	3.2	1.3–7.5	0.009
Temperature Day 0 (°C)	1.5	1.1–2.2	0.020

SP: sulphadoxine-pyrimethamine

AS/SP: artesunate/sulphadoxine-pyrimethamine

**Figure 4:**
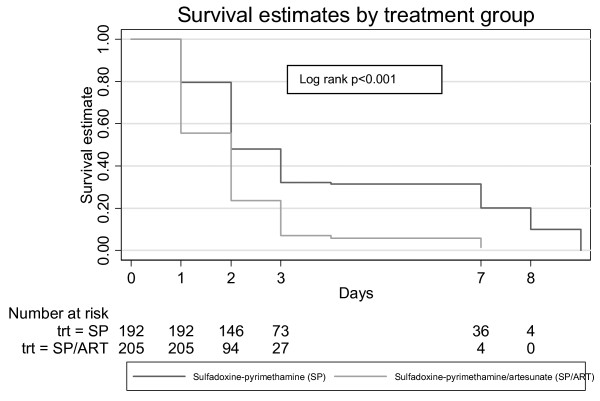
**Kaplan-Meier survival curves for time to parasite clearance**.

The ACT group had faster parasite clearance rates compared to the SP monotherapy group (log-rank p = 0.001) (Figure 4). Multivariate analysis (Table 5) found that ACT increased the rate of parasite clearance by 80% compared with monotherapy. In addition each 10-fold increase in baseline parasite density decreased parasite clearance by 20%. Subjects from Catuane cleared parasites 80% quicker compared to those from other sites. A significant interaction between treatment and parasite density suggested a faster parasite clearance by the ACT compared to monotherapy at higher parasite densities (HR 1.2; 95% CI 1.0–1.5 p = 0.033). However, the relative hazard of clearing parasites was similar for both treatment groups when modelled separately, indicating our overall results hold true for all subjects. The proportional hazard assumption was satisfied overall and for the treatment and Catuane effects. The baseline parasite density, however, was found to amplify the parasite clearance time by approximately 30% for every 10-fold increase. When the analysis was repeated excluding subjects with dose-related protocol violations, or censoring at the time of other violations, results were similar although there were no interactions identified, and age over seven years (HR 1.3: 95% CI 1.0–1.7 p = 0.046) was predictive of better parasite clearance compared to younger age.

**Table 5:** Multivariate model for the relative risk of parasite clearance

Variable	*Hazard ratio*	*95% CI*	*P*
Treatment with AS/SP vs. SP	1.8	1.4 – 2.3	<0.001
Log_10 _parasite density (per μl) blood)	0.8	0.7 – 0.8	<0.001
Catuane (compared to other sites)	1.8	1.3 – 2.5	<0.001

SP: sulphadoxine-pyrimethamine

AS/SP: artesunate/sulphadoxine-pyrimethamine

## Discussion

The aim of this study was to provide evidence to Mozambican health policy-makers regarding efficacy of SP with or without AS as treatment for uncomplicated malaria. In a survival analysis, both drug regimens were effective, however adding artesunate improved outcomes significantly with regard to clinical and parasitological cure, and peripheral parasite clearance time. These results are consistent with the literature, that suggests combining AS with SP improves efficacy, provided efficacy of SP is sufficiently high [7]. In addition, AS combined with SP showed an efficacy above 95%, the minimum level recommended by the WHO for policy implementation. The increased parasite clearance by the ACT corroborates data reflecting the higher parasite reduction ratio of the artemisinins (approximately 10^3^-10^5 ^parasites per asexual life cycle, compared to 10-10^3 ^for SP) [3].

Subjects aged over seven years had a 70% lower risk of treatment failure than those seven and under, and were 30% more likely to clear parasites faster (the latter association was only found when protocol violators were removed). These findings are consistent with the knowledge that immunity to malaria infection increases with age [22]. However, this protective effect is not fully understood as there is no clear immunological marker with which to validate it, and risk factors for malaria are entwined due to the vector, host and parasite relationships [22-24]. Another possible explanation for associations between lower age and poorer treatment outcome is that drug disposition in children may be altered leading to concentrations below the therapeutic threshold; physiological concentrations of anti-malarials at mg/kg labelled doses, particularly SP, may be sub-optimal in younger patients [25,26]. Dosing regimens derived from studies recruiting adults may lead to inappropriate recommendations for children particularly if regimens are age-rather than weight-based, although the former is more practical in resource-poor environments [27]. Conversely, adults may be under-dosed if they weigh more than 65 kg [28]. Bearing these findings in mind, healthcare providers should be trained to monitor children closely, in particular, for treatment failure.

Sulphadoxine and pyrimethamine are anti-folates that inhibit the dihydropteroate synthetase (*dhps*) and dihydrofolate reductase (*dhfr*) enzymes respectively. Resistance to SP develops due to accumulation of point mutations in genes that encode these enzymes. The presence of 5 mutations, 3 in the *dhfr *gene (S108N/N511/C59R) and 2 in the *dhps *gene (A437G/K540E) are associated with treatment failure [29,30]. This study found presence of the quintuple mutation was associated with a more than 3-fold increase in relative hazard of treatment failure compared to fewer mutations. Similar observations elsewhere have motivated proposals that molecular markers of resistance be used to predict future therapeutic response at the population level due to the myriad factors playing a role in treatment response [31]. The unexpected improvement in SP monotherapy cure rates between 2002, and this study's findings, may be explained in the decreased frequency of quintuple mutations seen in this province after the replacement of SP monotherapy with artemether-lumefantrine policy in neighbouring KwaZulu-Natal (KZN) [10]. However, this mutation frequency is expected to have increased again markedly since the study was concluded as SP containing regimens have been implemented country-wide for both the 1^st ^line treatment of uncomplicated malaria (AQ-SP replaced with AS-SP) and as intermittent preventive treatment for pregnant women (SP in 2007).

This study showed a significant association between higher temperature at baseline and greater relative hazard of treatment failure, as found in other studies [32]. The biological mechanism for this association is unclear, and temperature fluctuations, the concomitant use of pyretics for malaria and the presence other febrile conditions all indicates body temperature is an inconsistent predictor of outcome.

Higher parasitaemia at baseline was not found to be an independent risk factor for treatment failure, as opposed to findings in other studies, although it was associated with delayed parasite clearance [33]. The interaction observed between treatment and parasite density suggested the ACT effect was amplified at higher parasitaemias, as would be expected given the specific indication for artemisinin derivatives in uncomplicated hyperparasitaemias [34]. However, this interaction did not translate into a clear difference between treatments.

This study was able to achieve reasonably good subject retention and completeness of data in resource-poor community settings, which have inherent migration risk factors. Standardized training and methodologies for outcomes' measurements together with quality control measures, especially those relating to parasitological end points, minimized the possibility of measurement error. While the study was open-label after concealed allocation, the primary end point was measured by staff blinded to treatment group. Recrudescence occurred beyond 42 days, albeit in small numbers, validating this minimum recommended schedule and suggesting that, should resources allow, follow up be extended further [35].

When data were re-analysed excluding 57 (13.9%) protocol violators, results were very similar. Including data from non-adherent subjects or those taking prohibited concomitant medications is useful for assessing the effect of these common practices on treatment response. AS courses shorter than three days are not recommended due to their lower efficacy and the increased likelihood of anti-malarial drug resistance emerging if combined with the more slowly eliminated SP [5,6]. SP may, however, be sufficient to successfully treat malaria in individual subjects who take a substantially reduced dose of AS should its own efficacy be high enough. The study showed that in 90% of subjects SP monotherapy was able to effect an ACPR. The retrospective addition of erythromycin as a prohibited drug meant it was the predominant contributor to this subset of violations (36/43 [83.7%]), however there was no evidence of erythromycin playing a role in curing uncomplicated malaria or improving parasite clearance.

## Conclusion

This study found that, while both SP monotherapy and in combination with AS were effective, the ACT was far superior, supporting its current use in Mozambique as the national policy for the treatment of uncomplicated malaria. However, it is recognized that combinations with SP may have a limited useful life due to the spread of parasite resistance, and inadequate drug levels young children achieve. It is also possible that SP efficacy has declined since this study due to the national implementation of SP as an intermittent preventive treatment policy for pregnant women during 2007. Of further concern is the presence of the *dhfr *164 mutation, associated with high-level pyrimethamine resistance found in neighbouring Malawi [36].

## Competing interests

The authors declare that they have no competing interests.

## Authors' contributions

EA participated in study design, coordinated the study, conducted the statistical analysis and drafted the manuscript, TC managed the Mozambique study teams, YC provided medical expertise to the study teams, FL participated in study design, calculated sample sizes and supervised the statistical analysis, JR conducted molecular analyses, AB supervised analysis and reporting. KIB, Principal Investigator of the South East African Combination Anti-malarial Treatment (SEACAT) Evaluation, conceived of, and designed the study, and contributed to the analysis. All authors read and approved the final manuscript.
